# Novel tensorial Thixo-Visco-Plastic framework for rheological characterization of human blood

**DOI:** 10.1038/s41598-021-01362-8

**Published:** 2021-11-09

**Authors:** André Pincot, Matthew Armstrong

**Affiliations:** Department of Chemistry and Life Science, Chemical Engineering Program, United States Military Academy, West Point, NY 10996 USA

**Keywords:** Biological techniques, Biotechnology, Physiology, Biomarkers

## Abstract

Characterizing human blood, a complex material with a spectrum of thixo-elasto-visco-plastic properties, through the development of more effective and efficient models has achieved special interest of late. This effort details the development a new approach, the tensorial-enhanced-Thixo-Visco-Plastic model (t-e-TVP), which integrates elements from the proven Bingham and generalized Maxwell systems to create a more robust framework and subsequently cast into a tensorial format. Here, the elastic and viscoelastic stress contributions from the microstructure are superimposed upon the viscoelastic backbone solution for stress offered by the modified TVP frame. The utility of this novel model is tested against the contemporary tensorial-ethixo-mHAWB (t-ethixo-mHAWB) framework, a similar model with a greater number of parameters, using rheological data of human blood collected on an ARESG2 strain-controlled rheometer. The blood samples are parametrically and statistically analyzed, entailing the comparison of the t-e-TVP and t-ethixo-mHAWB models with their capacity to accurately predict small and large amplitude oscillatory shear as well as unidirectional large amplitude oscillatory shear flow in blood.

## Introduction

Fluids such as human blood demonstrate several non-Newtonian properties that can render classic rheological models insufficient for effective analysis. The unique nature of blood’s viscosity and elasticity is in large part due to the fluid’s suspended microstructures such as red blood cells, white blood cells, and platelets. These collectively lead blood to demonstrate the shear-thinning, viscoelastic, yield stress, and thixotropic behaviors characteristic of complex materials. Importantly, red blood cells (RBC) present the predominant portion of the microstructures suspended in the blood plasma and are thus of utmost significance in affecting blood’s fluid properties. RBCs have been shown to agglomerate into coin-stack like structures through fibrinogen fusing at low shear rates^1,2^. In observing this shear-dependent formation and breakdown phenomena of these rouleaux, the thixotropic nature of blood becomes further evident. In such fluid, complex materials, microstructure such as rouleaux can interact with the solvent medium to dramatically alter the behavior of the material.

The status of blood as a thixo-elasto-visco-plastic (TEVP) fluid entails notable differences in the methods by which its peculiar rheological behavior might be analyzed^3^. A notable feature of some TEVP materials is shear thinning, describing a tendency for fluid viscosity to decrease under mounting shear stresses^4,5^. This phenomenon can be observed in the disintegration of blood’s rouleaux microstructures, lending the fluid a more free manner of flow^6^. A comprehensive understanding of shear thinning is necessary to better characterize its behavior in natural, necessary behaviors such as blood clotting, where blood viscosity increases dramatically near wounds to seal breaches in the circulatory system. In addition to body health and blood composition, certain pharmaceuticals have been observed to affect blood shear properties. The evolution of this shear thinning can potentially induce stress in the vascocirculatory system as the body attempts to maintain a state of equilibrated flow throughout^1,3,5,7,8^.

As a TEVP material, blood also possesses a yield stress that ensures that its cells do not deform with some degree of stress application, were Newtonian fluids would, conversely, be inclined to deform without consistent application of a preserving force. This complex behavior entails that the cardiovascular system must continually apply work to maintain the blood yield necessary for healthy flow^6^. Relevant to this effort, ex-vivo experimentation on work-induced fluid deformation properties can be prosecuted via the use of a rheometer^7^. In addition to blood’s inherent material properties, the present microstructure can contribute to total yield stress characteristics. This relates to the thixotropic aspect of TEVP materials such as blood, which describes the evolution and devolution of the rouleaux microstructure at low and high shears respectively^7,9^. The elastic component refers to blood’s pre-deformation elastic properties^8,10,11^. The last component, plasticity, hails to the plastic nature of the rouleaux, which can deform and given a certain degree of applied shear, undergo irreversible change^4,5^.

In the past years, classical steady-state modeling has undergone several evolutions, with the Casson, Carreau-Yasuda, Bingham, and Herschel-Bulkley models proving particularly notable frameworks. However, these simple models lack the ability to deliberately and accurately characterize the evolving nature of the steady-state and transient flow regimes that are present in TEVP materials such as blood. The thixotropic aspect of complex materials generally acts to reduce the adherence of the model to collected data, especially at lower strain amplitudes. This fact resulted in the development of a new generation of models integrating systems of timescale-based differential equations and constraints to better describe the nuances of TEVP fluids^1,3,4,8,12–23^.

While initial enhanced models were simply classical models outfitted with additional functions and parameters, models such as that proposed by Dullaert and Mewis completely novel methods by which to analysis complex materials, grounded within the peculiar physical processes that distinguish TEVP fluids. Shared by these models and other Maxwellian are three primary thixotropic features: shear breakage representing the dissolution of microstructure like blood rouleaux, shear aggregation describing the inter-microparticle interactions of a fluid at a certain shear rate, and Brownian aggregation. The Brownian aggregation term aims to somewhat characterize the way RBCs spontaneously aggregate due to the random nature of Brownian motion^3,4,12^.

The thixotropic components of these novel models generally utilized three modes of structure dynamics: shear structure breakage, structure reconstitution due to Brownian forces, and shear aggregation^1,3,4,8,14–23^. Some newer thixotropic models, such as that developed by Wei and Solomon, incorporated these structure dynamics, incorporating a function for back-stress derived from the material microstructure^24,25^. In more accurately representing the formation and dissolution of microstructure with varying shear rate, such models can more accurately model microstructure under steady-state and transient flow regimes. In these models, the structure parameter, λ, describes a fully structured material at a value of 1 where each particle enjoys a full range of connections with its neighbors. However, as λ approaches 0, the microstructure decays^14,15,21^. This parameter allows for a more comprehensive representation of the microstructure deformation that can occur in complex materials with changing shear rate. This deformation leads to viscoelastic activity within the material, entailing a measurable stress response^3,8,15,21,22,26^. The model’s elasticity calculation also changes with this evolution in structure, necessitating correction of the model’s yield stress and elastic modulus^16,17,26^. As such, the total stress is the sum of that of the solvent structure and microstructure. Additionally, the model defines two types of viscosity in a flow state: viscosity due to variable microstructure λη_ST_ and pure solvent viscosity η_∞_^14,15^. By accounting for the various elements of the total viscosity, the model can more effectively represent viscosity evolution under transient shear rate conditions^14,15^.

Furthermore, blood’s viscoelasticity necessitates the depiction of microstructure-dependent elasticity via dual elastic and plastic stress components or, alternatively, the viscoelastic model’s inclusion of the structure parameter^17,18^. The former method involves the separation of the total strain and its time derivative into two, independent functions^12,17,21^. The kinematic hardening theories of plasticity, encompassing isotropic hardening (IH) and kinematic hardening (KH) can be applied to model plastic behavior, can be applied to modeling a material’s plastic behavior. The IH is relevant not just to plasticity but also the material’s thixotropic properties, as described by a dimensionless, internal structure parameter. KH describes the effective yield stress as a function of deformation and can induce delays between back stress evolution and shear stress^5,12,41^. Inclusion of IH and KH in modeling TEVP systems is vital though not necessarily sufficient for a comprehensive model of viscoelastic behavior, necessitating the addition of thixotropic structure parameters, kinetic equations, and viscoelasticity.

The separation of thixotropic response, viscoelastic response, shear structure breakage and structure build-up into separate timescales in models such as ethixo-mHAWB best allows the accurate replication of blood’s rheological behavior and the rouleaux within^3,44^. While this does add more parameters, contributing to model complexity, the new thixotropic and viscoelastic timescales provide vital insight into the evolution of the bloodstream’s rouleaux. CFD modeling can then be used in conjunction with such enhanced models for more effective blood analysis.

While a basic depiction of kinematic hardening had been present in earlier thixotropic models, the ability to test the comprehensiveness of newer TEVP models was hamstrung by a relative lack of detailed transient experimental data. However, as more rigorously collected data, has become available in recent years, progress on the further development of thixotropic models has become possible^1,8^. This new data extended beyond that of the steady-state, including variable amplitude and frequency datapoints from UD-LAOS analysis, drawn from the superposition of steady and oscillatory shear of the thixotropic model. This best allows for the representation of the RBCs’ viscoelastic feature via the use of a generalized White–Metzner-Cross model, producing a thixotropic viscoelastic model (TVM or, formerly, HAWB)^8^. The improved mHAWB variant of the model would manifest through the incorporation of a rouleaux viscoelastic response into TVM^3^. The mHAWB model itself has seen several modifications and improvements, with new elements integrated to produce the ETV and ESSTV models^3,44^.

Recent research has extended to the exploration of different models, with Armstrong and Tussing and Armstrong and Pincot investigating the potential use of the Oldroyd-8 and Giesekus models, respectively, to describe RBC behavior as an alternative to the ubiquitous generalized White–Metzner-Cross framework as used in the mHAWB model^35–38^.However, other research by Armstrong et al. delved into the potential use of Saramito’s Herschel-Bulkley model to better portray blood rheology while discounting the viscoelastic nature of deformable RBCs to produce the ethixo elastoviscoplastic (ethixo EVP) model. The ethixo EVP model integrated thixotropy trough the inclusion of a structural parameter bound to a kinetic equation to better represent the complexity of TEVP fluid^39^. The addition of thixotropy was shown to better the fit the model to the transient experimental blood rheological data.

A different approach taken in adding viscoelastic features to established viscoplastic and thixotropic models also entailed a better fit to the collected data^1,5^. As developed by Wei et al., the ML-IKH model provided an array of lambda values, featuring independent thixotropic evolution timescales, parallel to an isotropic kinematic hardening framework^40^. An analogous effort was accomplished in the modification of the SPTT-Isotropic Kinematic Hardening model (“*S”* for Saramito’s novel plasticity term, *“PTT”* for the Phain-Thien Tanner viscoelastic model) which acted to combine several approaches to effectively representing the material physics of a TEVP materal^17,41^. These models possessed a tensorial form and possessed 11 to 15 distinct parameters. Concurrent to the developments, the Modified Delaware Thixotropic Model (MDTM) was enhanced with a viscoelastic timescale of the stress response contribution from the component rouleaux, the novel model being dubbed viscoelastic enhanced MDTM (VE-MDTM)^23,31,35,42,43^. This alteration further demonstrated the importance of including the stress response from changes in microstructure. Recent work has also extended toward the tensorial transformation ETV and ESSTV model into tensorial analogues: t-ETV and t-ESSTV respectively^45^.

Despite the relative effectiveness of current generation models in fitting steady state and, most notably, transient rheological data for blood, their relative efficiency in analyzing the material nature of blood does leave something to be desired^5,10^. This effort utilizes elements from the previously established steady-state variants of the Dullaert & Mewis and MDTM models. These are then recast into a dynamic Maxwellian format, allowing for a tensorial representation^17,41^. The development of this new system, dubbed the tensorial-enhanced-Thixo-Visco-Plastic (t-e-TVP) model, is fully described in “Model development” section, where the new method is shown to integrate theories of plasticity to better express the elastic and viscoelastic contributions of the blood rouleaux towards total stress and integrate the full stress tensor^17,40,41^. “Methods” section follows with a description of the experimental protocol relevant to the collection of the experimental samples and a walkthrough of the parametric optimization performed to fit the experimental model to the given data. “Results and discussion” section includes a summary of the experimental results, analyzing the capabilities of the model to predict large amplitude oscillatory shear and uni-directional large amplitude oscillatory shear flow in the circulatory system. The accuracy of the novel t-e-EVP framework in predicting SAOS, LAOS, and UD-LAOS is then compared to that the t-ethixo-mHAWB variant, representing one of the most modern contemporary enhanced rheological modes. The conclusions of the analysis are then enumerated in “Conclusions” section.

## Model development

Being of Maxwellian origin, the steady state structure parameter of the tensorial-enhanced-Thixo-Visco-Plastic framework is defined as such:

1 $$ {\uplambda }_{{{\text{ss}}}} = \frac{{({\text{t}}_{{{\text{r}}2}} \left| {{\dot{\gamma }}} \right|^{{\text{d}}} + 1)}}{{({\text{t}}_{{{\text{r}}1}} \left| {{\dot{\gamma }}} \right| + {\text{t}}_{{{\text{r}}2}} \left| {{\dot{\gamma }}} \right|^{{\text{d}}} + 1)}} $$

where ${\text{ t}}_{{{\text{r}}1}}$ and ${\text{t}}_{{{\text{r}}2}}$ represent the ratios of rouleaux shear breakdown to Brownian buildup, and the ratio of shear aggregation to Brownian build-up respectively and ${\dot{\gamma }}$ is the fluid shear rate.

(For the tensorial form, ${\dot{\gamma }}$ is given as the second invariant of the rate of strain tensor: $\gamma_{(1)} = \dot{\gamma }_{{{\text{ij}}}} = \underline {\nabla } \underline {\nu } + \left( {\underline {\nabla } \underline {\nu } } \right)^{{\text{T}}}$). For this particular application of the steady-state structure parameter equation, the power law of shear aggregation, usually defined as *d*, is set to ½ per the findings of previously published literature on the subject of the blood medium^8,37,45^. As mentioned above, the steady-state thixotropy value is constrained between 0 and 1, with the former indicating the presence of no bonds between a given RBC and its neighbor and the latter signifying maximum structure agglomeration^14,15,21^.

To capture the elastic and viscoelastic stress contributions from the blood’s component rouleaux, we adhere to the approach of Horner et al. in integrating the following equation into the model, where the structural evolution manifests as a function of blood’s shear rate^3,8,37,45,46^:

2 $$ {\dot{\lambda }} = \frac{1}{{{\uptau }_{{\uplambda }} }}\left( { - {\text{t}}_{{{\text{r}}1}} \left| {{\dot{\gamma }}} \right|{\uplambda } + {\text{t}}_{{{\text{r}}2}} \left| {{\dot{\gamma }}} \right|^{{\text{d}}} \left( {1 - {\uplambda }} \right) + \left( {1 - {\uplambda }} \right)} \right) $$

where ${\uptau }_{{\uplambda }}$ is the total rouleaux agglomeration constant and, as before, *d* is set to ½ in deference to blood’s unique properties^8,37,45^. Equation (2) contains three separate components relevant to the thixotropic breakdown and formation of Rouleaux: 1) Shear breakdown proportional to shear rate and the amount of remaining structure, (2) Shear buildup characterized by $\left( {1 - {\uplambda }} \right)$; and (3) Brownian aggregation also defined by $\left( {1 - {\uplambda }} \right)$ ^3,46^. In capturing all three of these rouleaux phenomena, the model’s capability for concrete analysis is enhanced. Further, the generalized Maxwellian framework is evolved for use in defining the steady-state yield stress of the rouleaux, producing:

3 $$ {\upsigma }_{{{\text{ss}}}} = {\upsigma }_{{{\text{y}},0}} {\uplambda }_{{{\text{ss}}}} + {\upeta }_{{{\text{ST}}}} {\uplambda }_{{{\text{ss}}}}^{{\text{m}}} {\dot{\gamma }} + {\upeta }_{\infty } {\dot{\gamma }} $$

where ${\upsigma }_{{{\text{y}},0}}$ represents the yield stress, *m* represents an additional power law parameter, set to 3/2 in this work, while ${\upeta }_{{{\text{ST}}}}$ and ${\upeta }_{\infty }$ represent the structural viscosity and infinite viscosity respectively. The t-e-TVP’s fundamental description of the stress contributed by individual RBC’s possible deformations manifests as a modification and combination of the Kelvin-Voigt:

4 $$ {\upsigma }_{{{\text{y}},0}} = {\upgamma }_{{\text{e}}} {{G\lambda }} $$

and generalized Maxwellian:

5 $$  \frac{\eta }{{\text{G}}}\dot{\sigma } + \sigma  = \eta \dot{\gamma }  $$

yielding:

6 $$ {\dot{\sigma }} = \frac{{\text{G}}}{{{\uplambda }^{{\text{m}}} {\upeta }_{{{\text{ST}}}} + {\upeta }_{\infty } }}\left( {{{\lambda \sigma }}_{{{\text{y}}0}} + \left( {{\uplambda }^{{\text{m}}} {\upeta }_{{{\text{ST}}}} + {\upeta }_{\infty } } \right){\dot{\gamma }} - {\upsigma }} \right) $$

where G represents the elastic modulus. To better enhance the stability of the model equations, a tensorial approach is adopted in describing the individual *xx, yy*, *zz,* and *yx* axis components, the full Eqns. (7 – 10) shown below^3,8^:

7 $$ \left( {\frac{{{\uplambda }^{{\text{m}}} {\upeta }_{{{\text{ST}}}} + {\upeta }_{\infty } }}{{\text{G}}}} \right)\left( {{\upsigma }_{{{\text{xx}}}} - 2{\upsigma }_{{{\text{yx}}}} {\dot{\gamma }}_{{{\text{yx}}}} } \right) + {\upsigma }_{{{\text{xx}}}} = 0 $$

8 $$ \left( {\frac{{{\uplambda }^{{\text{m}}} {\upeta }_{{{\text{ST}}}} + {\upeta }_{\infty } }}{{\text{G}}}} \right){\upsigma }_{{{\text{yy}}}} + {\upsigma }_{{{\text{yy}}}} = 0 $$

9 $$ \left( {\frac{{{\uplambda }^{{\text{m}}} {\upeta }_{{{\text{ST}}}} + {\upeta }_{\infty } }}{{\text{G}}}} \right){\upsigma }_{{{\text{zz}}}} + {\upsigma }_{{{\text{zz}}}} = 0 $$

10 $$ \left( {\frac{{{\uplambda }^{{\text{m}}} {\upeta }_{{{\text{ST}}}} + {\upeta }_{\infty } }}{{\text{G}}}} \right)\left( {{\upsigma }_{{{\text{yx}}}} - 2{\upsigma }_{{{\text{yy}}}} {\dot{\gamma }}_{{{\text{yx}}}} } \right) + {\upsigma }_{{{\text{yx}}}} = {{\lambda \sigma }}_{{{\text{y}}0}} + \left( {{\uplambda }^{{\text{m}}} {\upeta }_{{{\text{ST}}}} + {\upeta }_{\infty } } \right){\dot{\gamma }}_{{{\text{yx}}}} $$

With the final introduction of the tensorial components, the description of the novel model is complete. As can be seen the model is fully capable of predicting the first normal stress difference, N1 as ${\upsigma }_{{{\text{xx}}}} {{ - \sigma }}{}_{{{\text{yy}}}}$. Table 1 contains a prompt summarization of the tensorial-enhanced-Thixo-Visco-Plastic model discussed here.

**Table 1:**
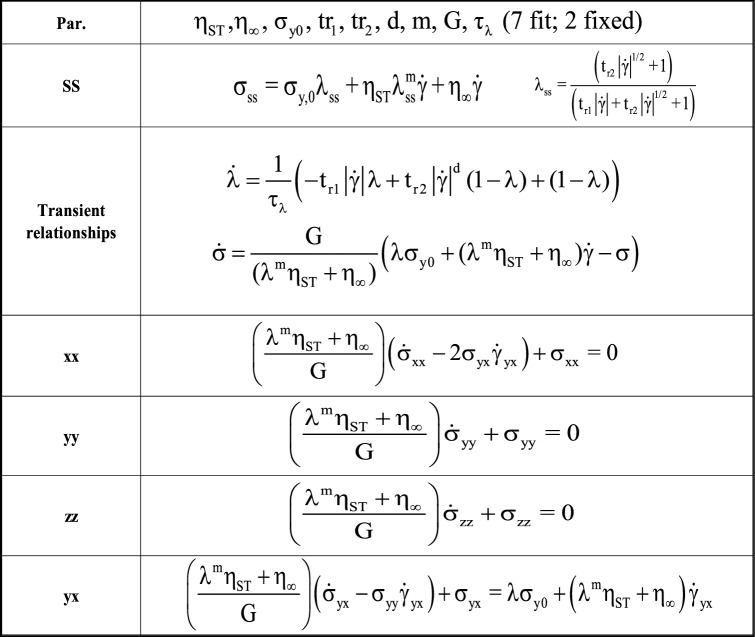
t-e-Thixo-Visco-Plastic Viscoelastic equations.

Being counted among one of the most useful contemporary models the tensorial ethixo-mHAWB, an enhanced tensorial variant of the original mHAWB model, provides a basis of comparison against for the framework developed above^3,44^. The t-ethixo-mHAWB approach requires 13 parameters, 10 of which must be fitted via the procedure discussed in “Methods” section. The model’s representation of viscoelastic stress contributions is shown below in Tables 2 and 3.

**Table 2:**
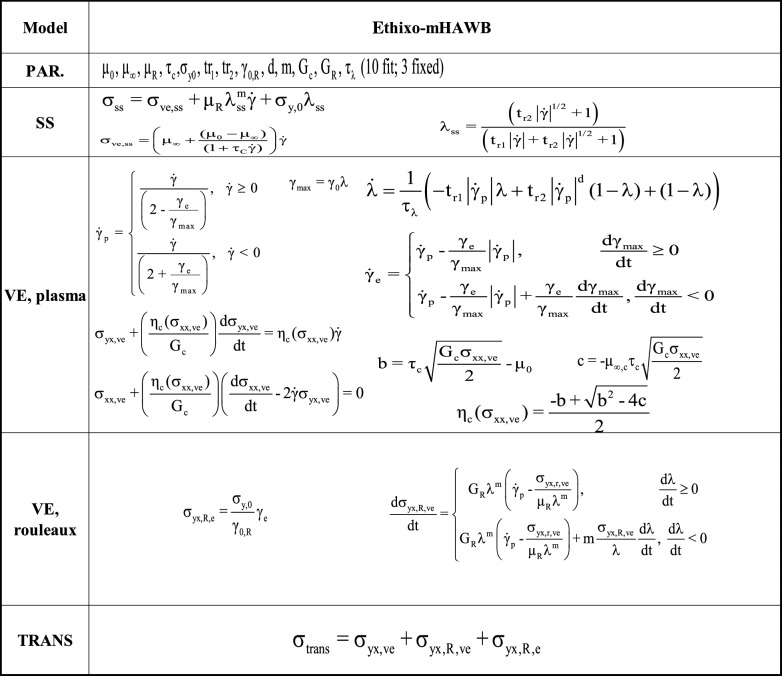
t-ethixo-mHAWB model Eqs. ^24,41^.

**Table 3:**
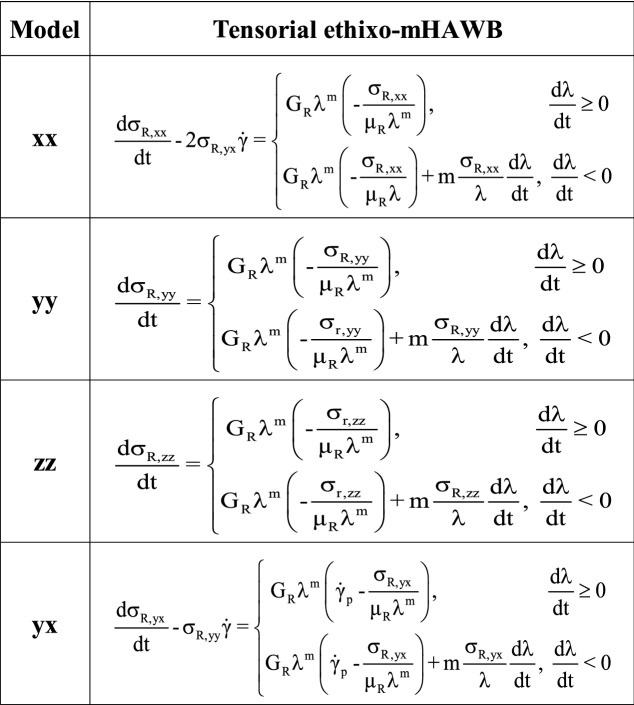
t-ethixo-mHAWB model equations (for the VE, rouleaux)^24,41^.

In utilizing the t-ethixo-mHAWB model for comparison, a clearer understanding of the relative efficacy of the new t-e-TVP model can be developed. The upcoming “Methods” section will detail the experimental procedure relevant to the procurement of the two analyzed blood samples and, subsequently, the methods by which the collected data was fitted to the two subject models.

## Methods

### Experimental protocol

The prosecution of this effort’s blood collection and rheological characterization protocols accord with existing precedent for blood rheology experiments, as previously enumerated by Horner et al^3,5,8,46^. The blood draw procedure was compliant with the expectations of the University of Delaware’s institutional review board, with the blood being drawn from the seated patients’ antecubital veins^3,5,8^. “The human blood samples were collected by a licensed practitioner at the Nurse Managed Primary Care Center located at University of Delaware STAR campus in *compliance* with, and approved by UD’s Institutional Review Board (Study Number 767478–2)^3–5^.” The methods were performed in accordance with our IRB’s relevant guidelines and regulations. Additionally, all human participants involved gave *informed consent* via face-to-face discussion, and signed documentation verifying such. At the time of the draw, the subjects had been confirmed to be free of any deleterious health conditions and had undergone fasting 8–10 h prior to the sample retrieval. 6 ml of retrieved blood was mixed with 1.8 mg/ml ethylenediaminetetraacetic acid within a vacutainer tube while 9 ml of blood was set aside for a comprehensive blood count, lipid panel, and fibrinogen activity test. The results of the latter testing can be found in Table 4 below^3,5,8^.

A TA Instruments ARES-G2 strain-controlled rheometer with a double wall couette was utilized for all experimental measurements^3,5,8^. The specific dimensions and measurable range of the couette geometry are identical to those of previous work^44,46,47^. No later than 60 min from collection the blood samples were introduced to the rheometer for testing, with all analysis occurring within 4 h from withdrawal. Through the tests the temperature was maintained at 37.0 °C via a Peltier temperature controller. Shear rate was consistently maintained below 1000 s^−1^ and each test was initiated with an applied preshear of 300 s^−1^ for 30 s to eliminate lingering memory effects on microstructure^3,5,8^.

Experimental results are shown for steady state, step up/down in shear rate tests, amplitude and frequency sweeps, large amplitude oscillatory shear (LAOS) and unidirectional large amplitude oscillatory shear flow (UD-LAOS). The set of procedures and protocols adhered to throughout the experimentation are identical to those detailed in preceding work by Horner et al. with all Table 1 rheological donor data available on Mendeley Data^49,50^. Additionally, the LAOS and UD-LAOS analysis adhere to methods established by Horner et al. and Armstrong et al.^3,4,8,35,37,39,46^. As such, experimental strain and shear rates are characterized as:

11 $$ \gamma \left( t \right) = \gamma_{0} {\text{sin}}\left( {\omega t} \right) $$

and

12 $$ \dot{\gamma }\left( t \right) = \gamma_{0} \omega {\text{cos}}\left( {\omega t} \right) $$

for LAOS and

13 $$ \gamma \left( t \right) = \gamma_{0} {\text{sin}}\left( {\omega t} \right) + t\gamma_{0} \omega $$

and

14 $$ \dot{\gamma }\left( t \right) = \gamma_{0} \omega {\text{cos}}\left( {\omega t} \right) + \gamma_{0} \omega $$

for UD-LAOS. Lissajous-Bowditch elastic and viscous progressions are used to then plot the data and model fits derived from the UD-LAOS analysis, depicting the measured stress as a function of the oscillatory component of the strain and shear rate shown in Eqs. (11) an (12) respectively^3,8,37,39,46^.

The representative function for both the amplitude and frequency sweep is as such:

15 $$ \sigma_{yx} \left( t \right) = \gamma_{0} \mathop \sum \limits_{i, odd}^{n} \left( {G_{i}^{^{\prime}} \sin \left( {n\omega t} \right) + G_{i}^{^{\prime\prime}} \cos \left( {n\omega t} \right)} \right) $$

where the function inputs are strain amplitude $\gamma_{0}$, time *t*, and frequency $\omega$. For the amplitude and frequency sweeps, the running index *n* is customarily set to 1, though the upper summation limit is infinite. Additionally, the primary metric of success for model predictions is found in the ratio of the third to first harmonic:

16 $$ \frac{{I_{3} }}{{I_{1} }} = \sqrt {\frac{{G_{3}^{^{\prime}2} + G_{3}^{^{\prime\prime}2} }}{{G_{1}^{^{\prime}2} + G_{1}^{^{\prime\prime}2} }}} $$

with the subscript on the elastic moduli representing the harmonic order^3,8,37,39^. Used to quantify the efficiency of a given model, Eq. (16) juxtaposes the relative intensity of the third harmonic to the first harmonic. This analysis of model effectiveness is shown on the models’ amplitude and frequency sweep predictions in Figs. 3 and 4^3,8,37,39^.

**Table 4:** Blood physiological parameters.

Donor	HCT	Fib	Total Chol	Tri	HDL	LDL
	(%)	(mg/dL)	(mg/mL)	(mg/mL)	(mg/mL)	(mg/mL)
1	38.3	0.333	145	144	39	82
2	45.2	0.214	187	57	65	108

### Parameter optimization

The parameter optimization process of the novel t-e-Thixo-Visco-Plastic model utilizes number of techniques derived from the work of Armstrong and Tussing^3,8,31,35,37,39,43,46^. To fit the model’s steady state parameters to the collected steady state data, the *normalized* cost function *F*_*cost,ss*_ must be minimized via the application of the parallel tempering algorithm below^3,8,37,39^:

17 $$ F_{cost,ss} = \frac{1}{N}\sqrt {\mathop \sum \limits_{i = 1}^{N} \left( {\frac{{\left( {y_{i} - f_{i} } \right)}}{{y_{i} }}} \right)^{2} } $$

where $$y_{i}$$ and $$f_{i} $$ represent the steady state stress data and model prediction respectively. The transient parameters are then experimentally fit to four tiers of shear rate step up and four tiers of shear rate step down with parallel tempering, acting to minimize the cost function:

18 $$ F_{cost,trans} = \frac{1}{M}\mathop \sum \limits_{i = 1}^{M} \frac{1}{N}\sqrt {\mathop \sum \limits_{i = 1}^{N} \left( {y_{i} - f_{i} } \right)^{2} } $$

where *M* and *N* are the total number of transient step up/down tiers in shear rate testing and number of datapoints respectively. The particulars of the experimental fitting procedure are like those of Armstrong at Tussing^35^. The rheological dataset predictions can be seen in Fig. 2 below, with LAOS and UD-LAOS cost functions calculated as such:

19 $$ F_{cost,LAOS} = \frac{1}{N}\sqrt {\mathop \sum \limits_{i = 1}^{N} \left( {y_{i} - f_{i} } \right)^{2} } $$

Amplitude and frequency sweep analysis is conducted with the cost function described as:

20 $$ F_{cost,Sweep} = \mathop \sum \limits_{i = 1}^{N} \left( {\sqrt {\left( {G_{i,data}^{^{\prime}} - G_{i,model}^{^{\prime}} } \right)^{2} } + \sqrt {\left( {G_{i,data}^{^{\prime\prime}} - G_{i,model}^{^{\prime\prime}} } \right)^{2} } } \right)/\left( {2n} \right) $$

where *n* represents the number of datapoint. $F_{cost,Sweep}$ is produced for both amplitude and frequency sweeps, while the model parameters are held constant to predict the full alternating period. This utilizes an array with ***A*** = ***bx*** where **x** consists of two elements: $\gamma_{0} {\text{sin}}\left( {\omega t} \right)$ and $\gamma_{0} {\text{cos}}\left( {\omega t} \right)$ while *b* is the period’s stress prediction^37,39^. The third harmonic moduli, $G_{3}^{^{\prime}}$ and $G_{3}^{^{\prime\prime}}$, are also derived as shown in Eq. (20). All of the parameter optimization for both models was conducted with Matlab, version R2021a, with a stochastic, global, parallel tempering algorithm. The data for the figures was obtained with Matlab, and the final figures were constructed with Origin Graphing and Analysis software version 2021b^31^.

The analysis of the LAOS and UD-LAOS integrate the experimentally derived $F_{cost,LAOS}$ over three data period, with the final period predictions and data demonstrated in the Lissajous-Bowditch elastic and viscous projections. The analysis procedure utilizes both the Akaike Information Criteria (AIC) and the Bayes Information Criteria (BIC) (with steady-state and transient step optimization) to juxtapose additional parameters against model accuracy, penalizing the addition of extra parameters^32,35,43,48^. The AIC and BIC residual sum of squares (RSS) is calculated as such:

21 $$ RSS = \mathop \sum \limits_{i = 1}^{N} \sqrt {\left( {y_{i} - f_{i} } \right)^{2} } $$

where $y_{i}$ and $f_{i}$ are the data and model prediction respectively. For AIC and BIC, the steady state cost function and RSS is normalized by^32,35,43,48^:

22 $$ AIC = 2k + 2{\text{ln}}\left( {RSS} \right) $$

23 $$ BIC = 2kln\left( n \right) + 2{\text{ln}}\left( {RSS} \right) $$

where *k* and *n* are the number of parameters and data points respectively^32,35,43^.

## Results and discussion

### t-e-TVP model

**Figure 1:**
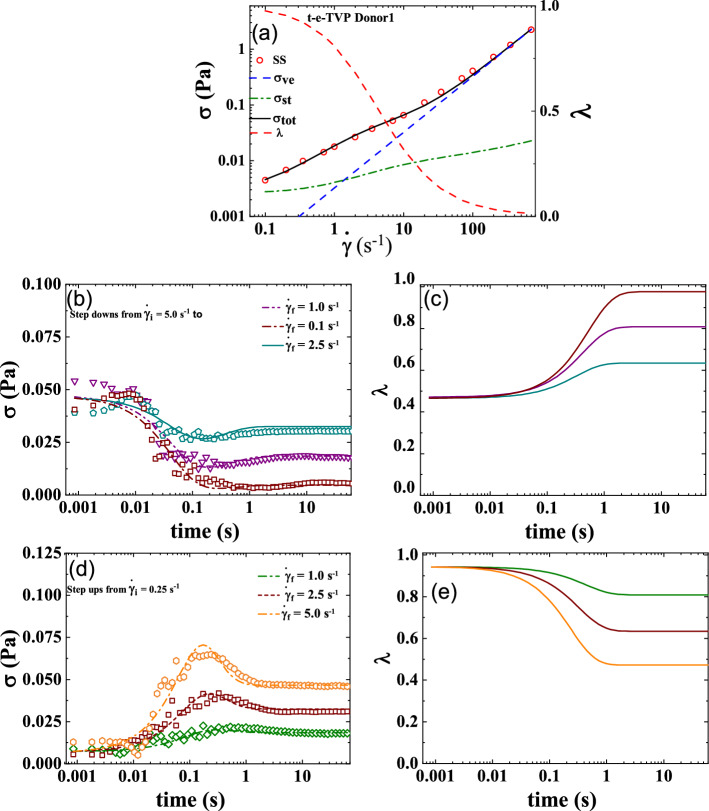
t-e-TVP fits to (**a**) steady state; (**b**) series of step downs from $\dot{\gamma }$ = 5 s^−1^ to 2.5, 1, 0.5 s^−1^; (**c**) representative structure parameter curves with colors corresponding to prior stress evolution curves; (**d**) step up in shear rate from $\dot{\gamma }$ = 0.25 s^−1^ to 1, 2.5, 5 s^−1^; and (**e**) representative structure parameter curves (Donor 1, Dataset 1)^49^.

The t-e-Thixo-Visco-Plastic framework possesses 9 total parameters, with two being fixed with reference values, five being fit with steady-state analysis, and the final two resulting from the transient step up/down shear rate analysis. The two fixed values, *d,* and *m* are set to ½ and 3/2 respectively, as previous literature recommends for analysis of blood^3,8,37,43,46^. Due to the tensorial nature of the novel model equations, a trivial, algebraic steady-state solution is unnecessary. As such, the transient and steady-state parameters are simultaneously fit to one set of steady-state data and six sets of transient step down/up shear rate data. Figure 1a details the steady state fit while Fig. 1b,d show the three iterations each of the step down/up transient shear rate fits. From these two plots, it can be deduced that the model is generally sufficient in predicting the stress under evolving shear rate of the experimentation. In addition, Fig. 1c,e showcase the corresponding structure parameter’s evolution as it is subjected to steps down and up in applied shear rate. The optimized fit model parameters from the steady-state and transient analysis, as well as the *F*_*cost*_ values for step down/up and amplitude/frequency sweeps, can be seen in Table 5. Table 5 also summarizes the model *AIC, BIC*, and *RSS* for Donor 1. Due to having been normalized at each data point, *F*_*cost,*_ (for the steady state) is a dimensionless value. Lastly, Fig. 3a–d show the model predictions of both the amplitude sweep performed at ω = 12.566 (rad/s), the frequency sweep performed at ${\upgamma }_{{0}} { = 10( - )}$, a series of UDLAOS projections, and a series of LAOS projections respectively. All figures shown are relevant to Donor 1^49^.

**Table 5:**
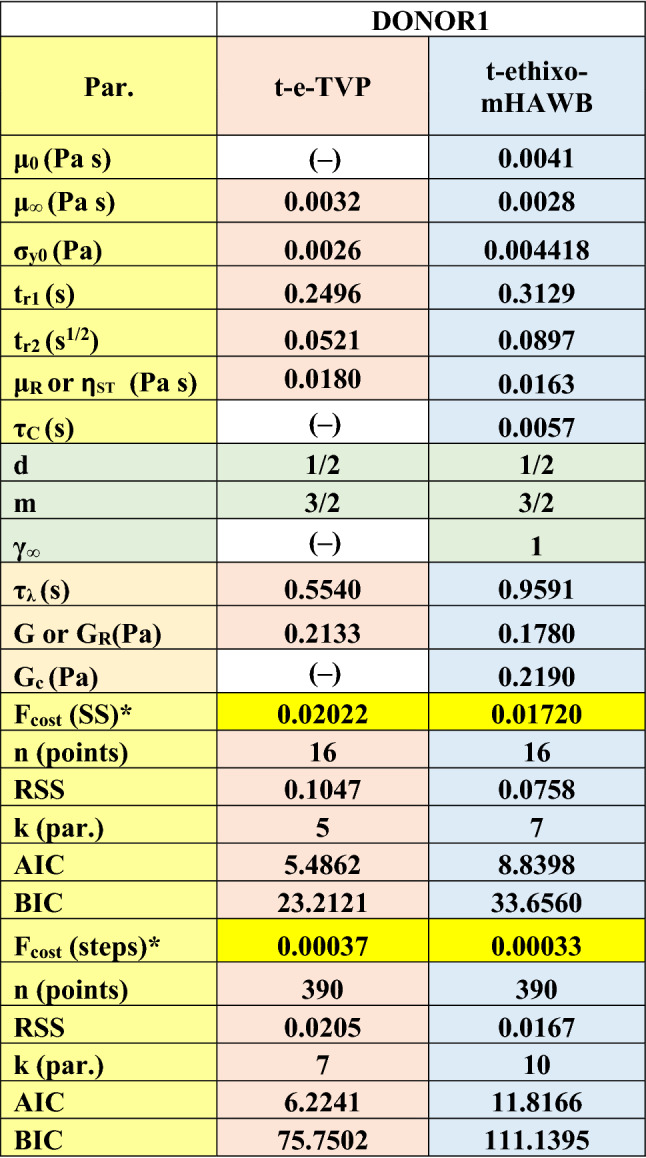
Best fit model parameters, and F_cost_ of fits for Donor 1 with t-e-TVP and t-ethixo-mHAWB models. Highlight legend: yellow–steady state fit; green—fixed; orange (salmon column) t-e-TVP step up/down fits; orange (blue column)—t-ethixo-mHAWB step up/down fits. (Donor 1, Dataset 1)^49^.

### t-ethixo-mHAWB

**Figure 2:**
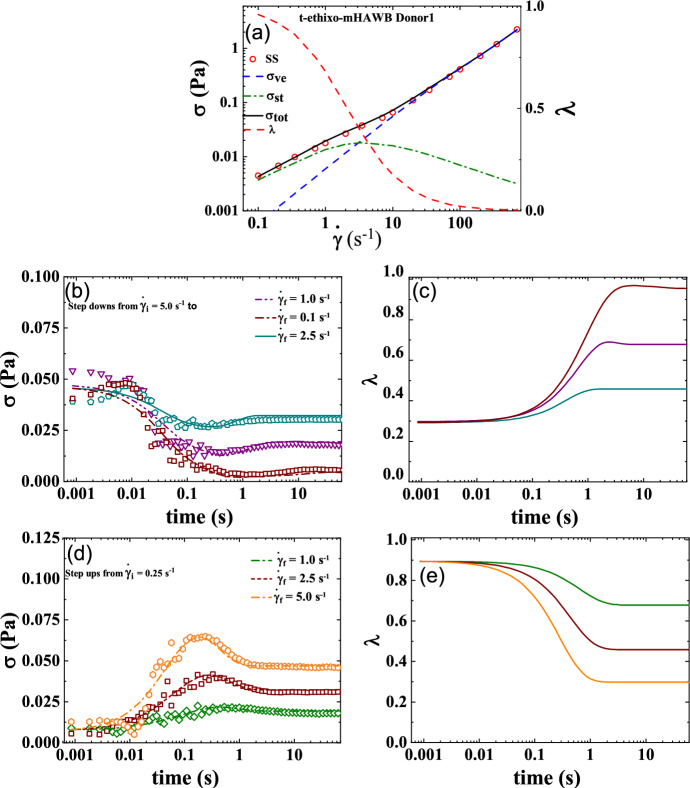
t-ethixo-mHAWB fits to (**a**) steady state; (**b**) series of step downs from $\dot{\gamma }$ = 5 s^−1^ to 2.5, 1, 0.5 s^−1^; (**c**) representative structure parameter curves with colors corresponding to prior stress evolution curves; (**d**) step up in shear rate from $\dot{\gamma }$ = 0.25 s^−1^ to 1, 2.5, 5 s^−1^; and (**e**) representative structure parameter curves (Donor 1, Dataset 1)^49^.

The t-ethixo-mHAWB model consists of 13 parameters, with three fixed with reference values, seven fit by steady-state analysis, and three from the transient step up/down shear rate analysis. As with the t-e-TVP approach, literature recommends the fixing of the two values *d* and *m* to ½ and 3/2 respectively^3,7,36,42,45^. As before, due the tensorial nature of the model equations prove a trivial, algebraic steady-state solution is unnecessary and the transient and steady-state parameters are fit simultaneously. Figure 2a details the steady state fit while Fig. 1b,d show the three steps of each of the step down/up transient shear rate fits. These two plots show that the t-ethixo-mHAWB is generally sufficient in predicting the stress under evolving shear rate of the experimentation and suitable for comparison with the experimental model. Figure 2c,e showcase the structure parameter’s corresponding evolution as it steps down and up in shear rate. The t-ethixo mHAWB best fit model parameters from the steady-state and transient analysis, *AIC, BIC, RSS*, and the *F*_*cost*_ values for step down/up, amplitude sweeps and frequency sweeps, can also be seen in Table 5. *F*_*cost,*_ is a dimensionless value due to having been normalized at each data point. Lastly, Fig. 4a–d show predictions for an amplitude sweep performed at ω = 12.566 (rad/s), and frequency sweep carried out at ${\upgamma }_{{0}}$ = 10 (−), UDLAOS projections, and LAOS projections respectively. As with the previous models, all figures shown draw from Donor 1 data^49^.

### Comparison of models

**Figure 3:**
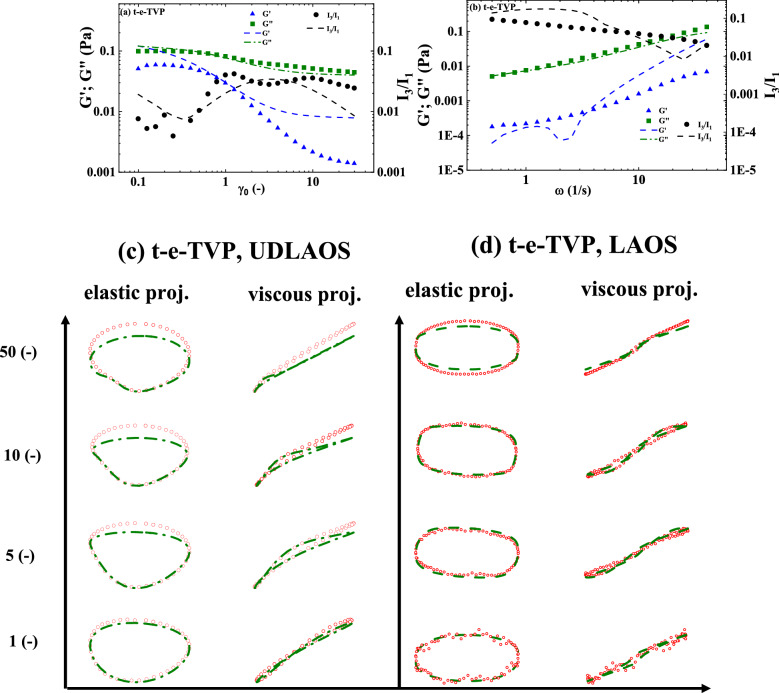
t-e-TVP prediction of (**a**) amplitude sweep executed at ω = 12.566 (rad/s); (**b**) frequency sweep executed at $\gamma_{0} = 10( - )$(**c**) elastic and viscous UDLAOS projections (green lines); and (**d**) elastic and viscous LAOS projections (green lines). (All UDLAOS and LAOS were conducted at ω = 1 (rad/s), and corresponding strain amplitude). Red dots indicate data; x-axis units are frequency (rad/s); y-axis units are strain amplitude (−). (Donor 1, Dataset 1)^49^.

**Figure 4:**
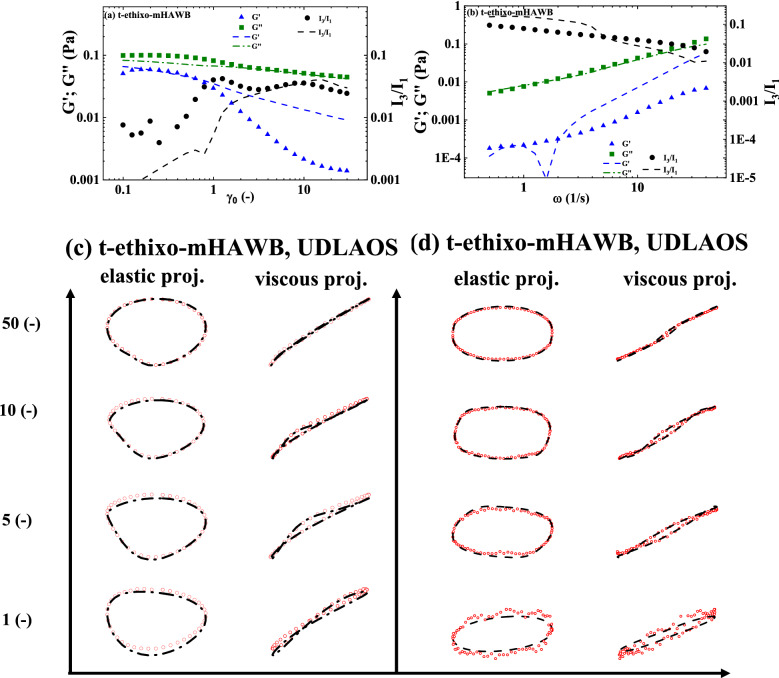
t-ethixo mHAWB prediction of (**a**) Amplitude sweep executed at ω = 12.566 (rad/s); (**b**) frequency sweep executed at $\gamma_{0} = 10( - )$; (**c**) elastic and viscous UDLAOS projections (green lines); and (**d**) elastic and viscous LAOS projections (green lines). (All UDLAOS and LAOS were conducted at ω = 1 (rad/s), and corresponding strain amplitude). Red dots indicate data; x-axis units are frequency (rad/s); y-axis units are strain amplitude (−). (Donor 1, Dataset 1)^49^.

As forestated, Tables 5 and 6 juxtapose relevant components of the steady state and transient fit cost functions, while exhibits (b) and (c) of Figs. 3 and 4 can be used to compare the LAOS and UDLAOS procedures between the t-e-TVP and t-ethixo-mHAWB models. A brief inspection of the averaged model F_cost, SS_, F_cost, Step_, F_cost, UDLAOS_, and F_cost, LAOS_ at the foot of Table 6 will reveal that there exists a tangible difference in the accuracy of the models. However, compared to other rheological models the performance of the t-e-TVP model proves satisfactory. In viewing Figs. 1b,d and 2b,d it is of note that both models’ predictive capability tends to suffer at higher applied shear rates, be it in step up or step-up shear rate scenarios. Further evidence of the novel model’s viability lies in a cursory inspection of components (b) and (c) of Figs. 3 and 4; interpretation of the elastic and viscous projection of LAOS and UDLAOS demonstrate the predominance of viscous effects, as is expected from the blood medium^8^. This interpretation is derived from the legends contained in Fig. S5 of the attached supplemental materials. Nonetheless, in this analysis it is important to remain cognizant that the demonstrated data is only that of Donor1, with figures for Donor 2 located in the supplemental materials. Figures 3a,b and 4a,b shows that both models perform nearly the same in prediction the storage and loss modulus, for the amplitude and frequency sweeps respectively. We note here that blood over this range of strain amplitudes and frequency always shows a larger value of loss modulus (liquid-like metric), G” than (solid-like metric) G’, as is expected, as human blood over this experimental range in shear rate, and temperature is more a liquid, than a solid.

**Table 6:**
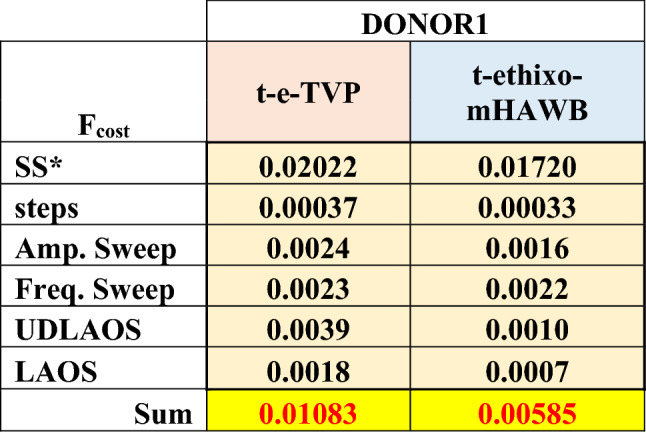
F_cost_ comparison for model predictions (Sweeps, LAOS, UDLAOS) over experimental fits (steady state, shear rate step tests; note that steady state is not included because it is nondimensionalized) (Donor 1).

For both Figs. 3a,b and 4a,b the G” values are predicted more accurately, than is G’ for both models, however the G’ trends are qualitatively predicted by both models. This is because human blood is challenging to work with since at most of the combinations of strain amplitude and frequencies the blood is much more ‘liquid-like’ than ‘solid-like’. This is reflected in the actual data and the model predictions. Furthermore, with respect to the amplitude sweep, to the left of values of strain amplitude: ${\upgamma }_{{0}} { = 1( - )}$*,* there is clearly some vestigial structure (rouleaux) present, while after this value there is not. On top of that, at the frequency of the amplitude sweep, *ω* = 12.566 (rad/s), and ${\upgamma }_{{0}} \le {1( - )}$*,* values less than this is in the vicinity of the linear region of SAOS. The ‘double-peak’ shown of the I_3_/I_1_ trend, most likely have to do with the fact that in this region there are rouleaux evolving: aka. breaking down and building up, whereby beyond this region values of strain amplitude and frequency are no longer conducive to rouleaux build-up.

Table 6 quantitatively demonstrates the same. In comparison of Figs. 3c and 4c (UDLAOS), and Figs. 3d and 4d (LAOS) we qualitatively see that both models’ predictive capability of UDLAOS and LAOS is almost identical until the point of strain amplitude equal or exceeding 10(−). At values of $\gamma_{0} \ge 10( - )$ the t-e-TVP is no longer able to quantitatively predict accurate stress values, while the t-ethixo-mHAWB can. This result is seen quantitatively in Table 6 and corroborated with Donor2 fits and predictions shown in the Supplemental Material. (Analogous fits and predictions for Donor 2 using both models are shown in the Supplemental Material).

## Conclusions

This effort has manifested in the development of a novel approach to the problems posed by TEVP fluids such as blood in the creation of a model derived from the modification and subsequent modification of the Kelvin-Voigt and generalized Maxwell frameworks. In applying precedent methods established in the work of Armstrong et al., Varchanis et al., and Wei et al., both modified models were enhanced with a full tensor, granting them relatively superior predictive performance under LAOS and UDLAOS conditions for both Donors 1 and 2 (see supplemental materials below)^39–41^. Moreover, this improvement merited the collection of no additional parameters. As has been found in previous literature, the improvements offered by the full tensor are marked^45^. While the predictive capabilities of the t-e-TVP model are not precisely equivalent to that of t-ethixo-mHAWB, analysis of both Datasets 1 and 2 indicated that the t-e-TVP model offers the benefit of drastically reducing the number of fitted parameters, possessing 9 total as opposed to its peer’s 13 total. This manifests as t-e-TVP’s considerably lower AIC and BIC values, offsetting the minor accuracy advantage of t-ethixo-mHAWB. Future efforts will likely pertain to the further optimization of the novel model’s performance through further modification as despite its relatively simple framework, necessitating few parameters, it offers considerable predictive potential. Possible future models will retain the full tensorial approach, with any considered parameter modification or additions grounded in the concrete mechanics of viscoelastic phenomena and microstructure evolution.

## Supplementary Information

Supplementary Information.
