# Supplementary material for: Novel tensorial Thixo-Visco-Plastic framework for rheological characterization of human blood
Source: Sci Rep. 2021 Nov 9;11:22004. doi: 10.1038/s41598-021-01362-8 (PMC8578634; doi:10.1038/s41598-021-01362-8)
Supplement: Supplementary file 1 — Supplementary Information. [file 41598_2021_1362_MOESM1_ESM.pdf]

Supplemental Material

Novel tensorial-enhanced- Thixotropic-Visco-Plastic Framework for Rheological Characterization of Human Blood

André Pincot, Matthew Armstrong

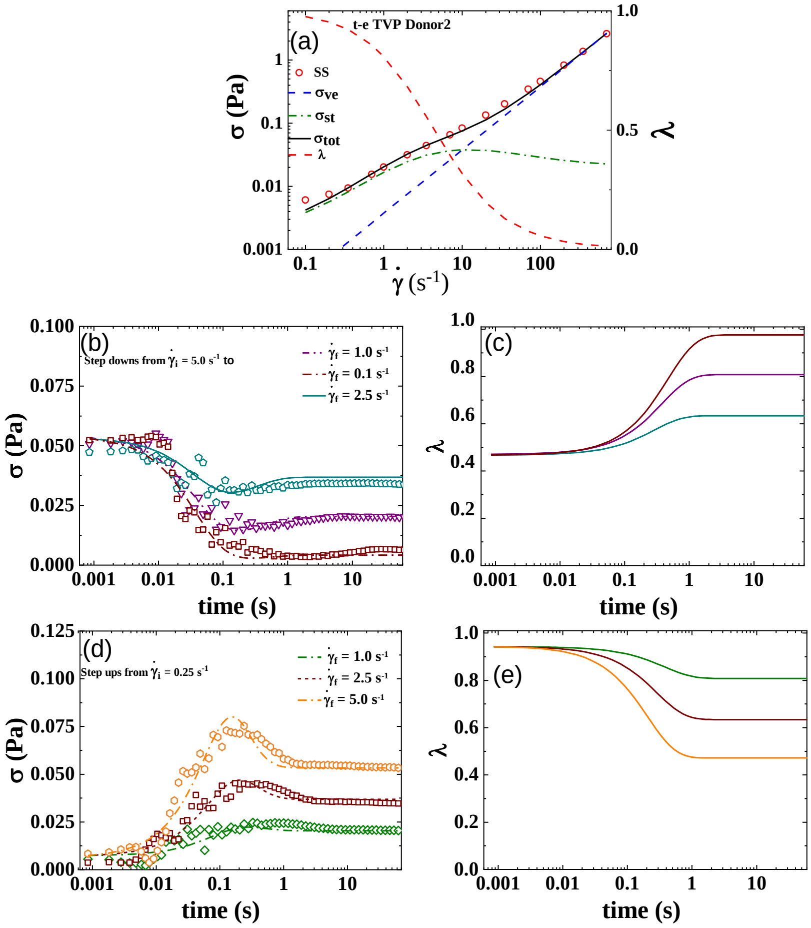

Figure S1. t-e-TVP fits to (a) steady state; (b) series of step downs from $\dot{\gamma}$=5s^{-1} to 2.5, 1, 0.5s^{-1}; (c) representative structure parameter curves with colors corresponding to prior stress evolution curves; (d) step up in shear rate from $\dot{\gamma}$=0.25s^{-1} to 1, 2.5, 5s^{-1}; and (e) representative structure parameter curves (Donor 2, Dataset 2).[50]

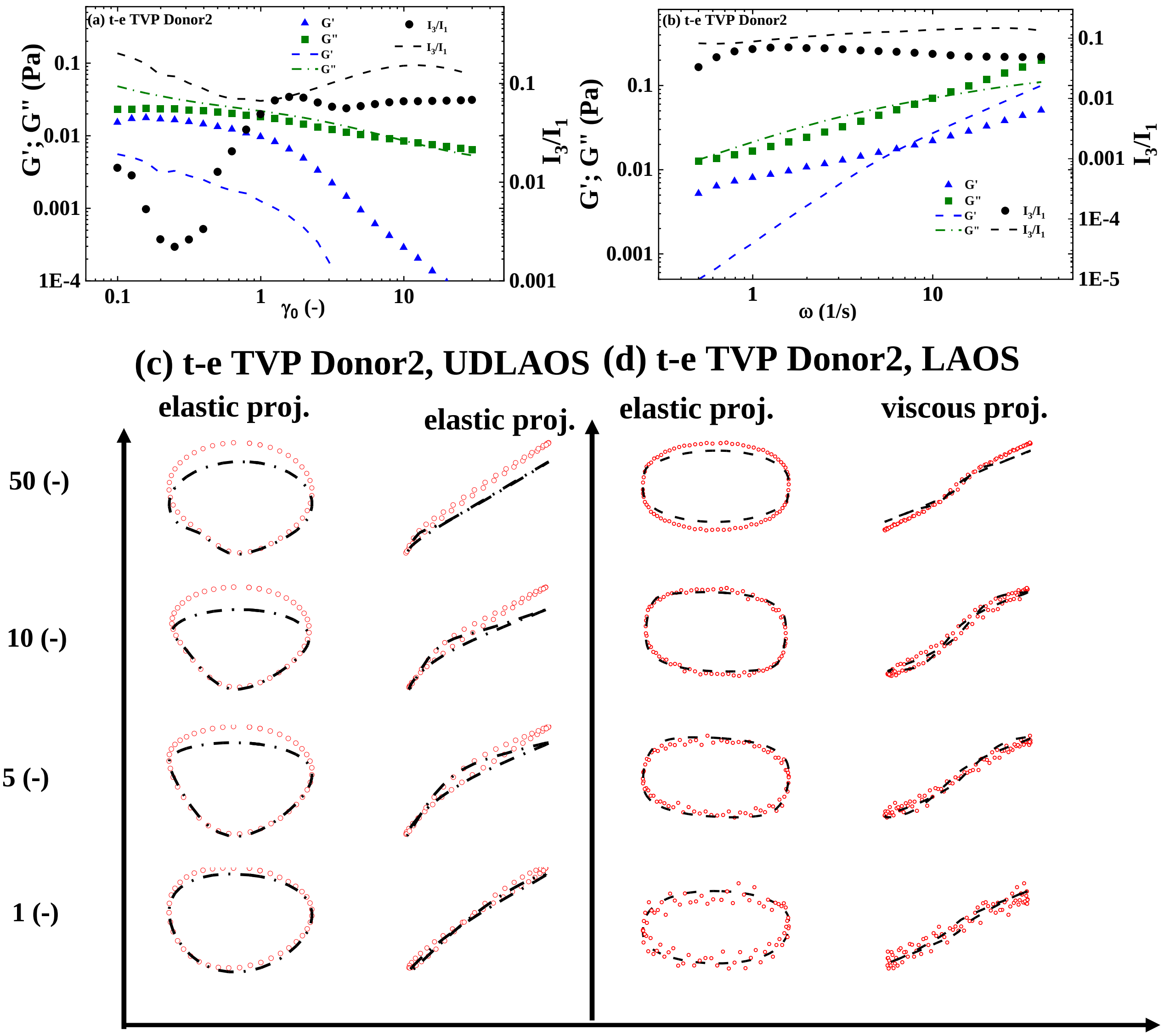

Figure S2. t-e-TVP prediction of (a) amplitude sweep executed at ω =12.566 (rad/s); (b) frequency sweep executed at γ_0=1(-); (c) elastic and viscous UDLAOS projections (green lines); and (d) elastic and viscous LAOS projections (green lines). (All UDLAOS and LAOS were conducted at ω =1 (rad/s), and corresponding strain amplitude). Red dots indicate data; x-axis units are frequency (rad/s); y-axis units are strain amplitude (-). (Donor 2, Dataset 2).[50]

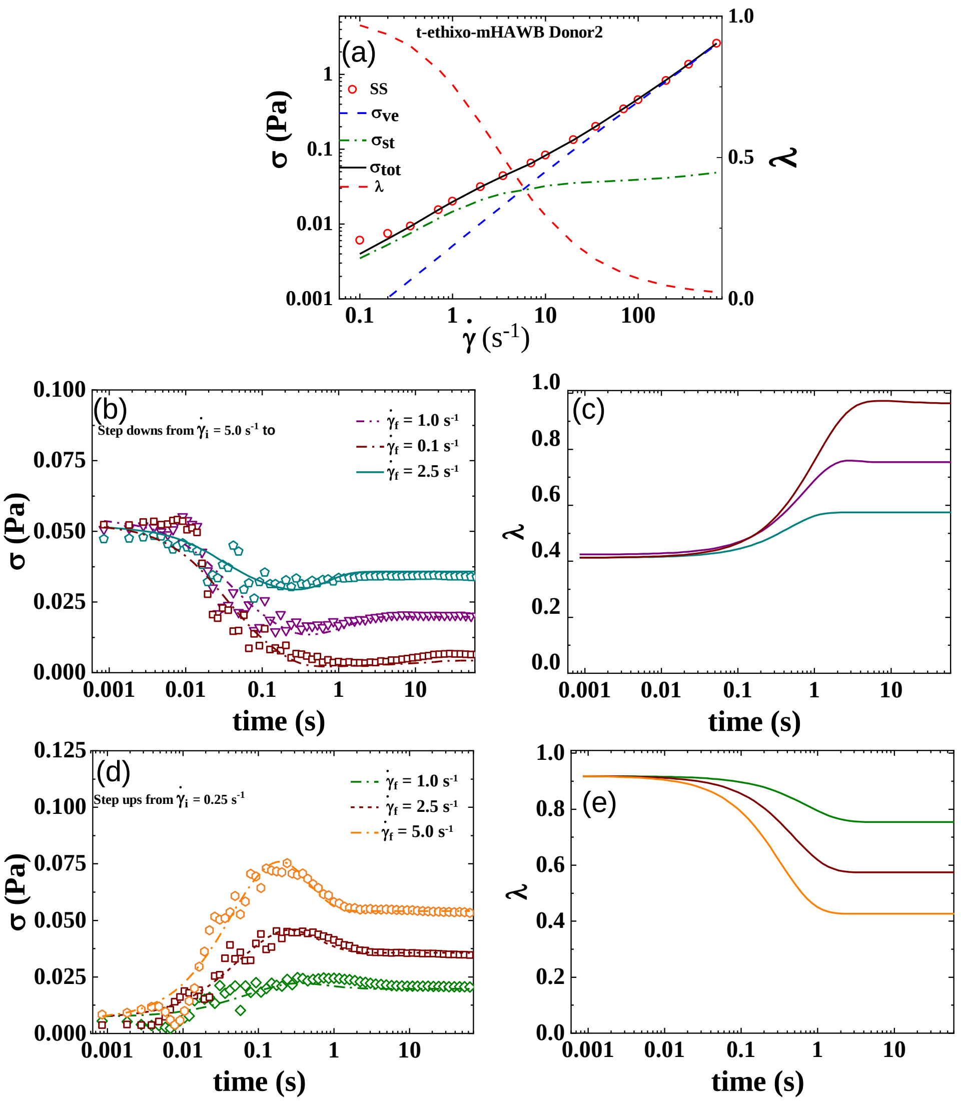

Figure S3. t-ethixo-mHAWB fits to (a) steady state; (b) series of step downs from $\dot{\gamma}$=5s^{-1} to 2.5, 1, 0.5s^{-1}; (c) representative structure parameter curves with colors corresponding to prior stress evolution curves; (d) step up in shear rate from $\dot{\gamma}$=0.25s^{-1} to 1, 2.5, 5s^{-1}; and (e) representative structure parameter curves (Donor 2, Dataset 2).[50]

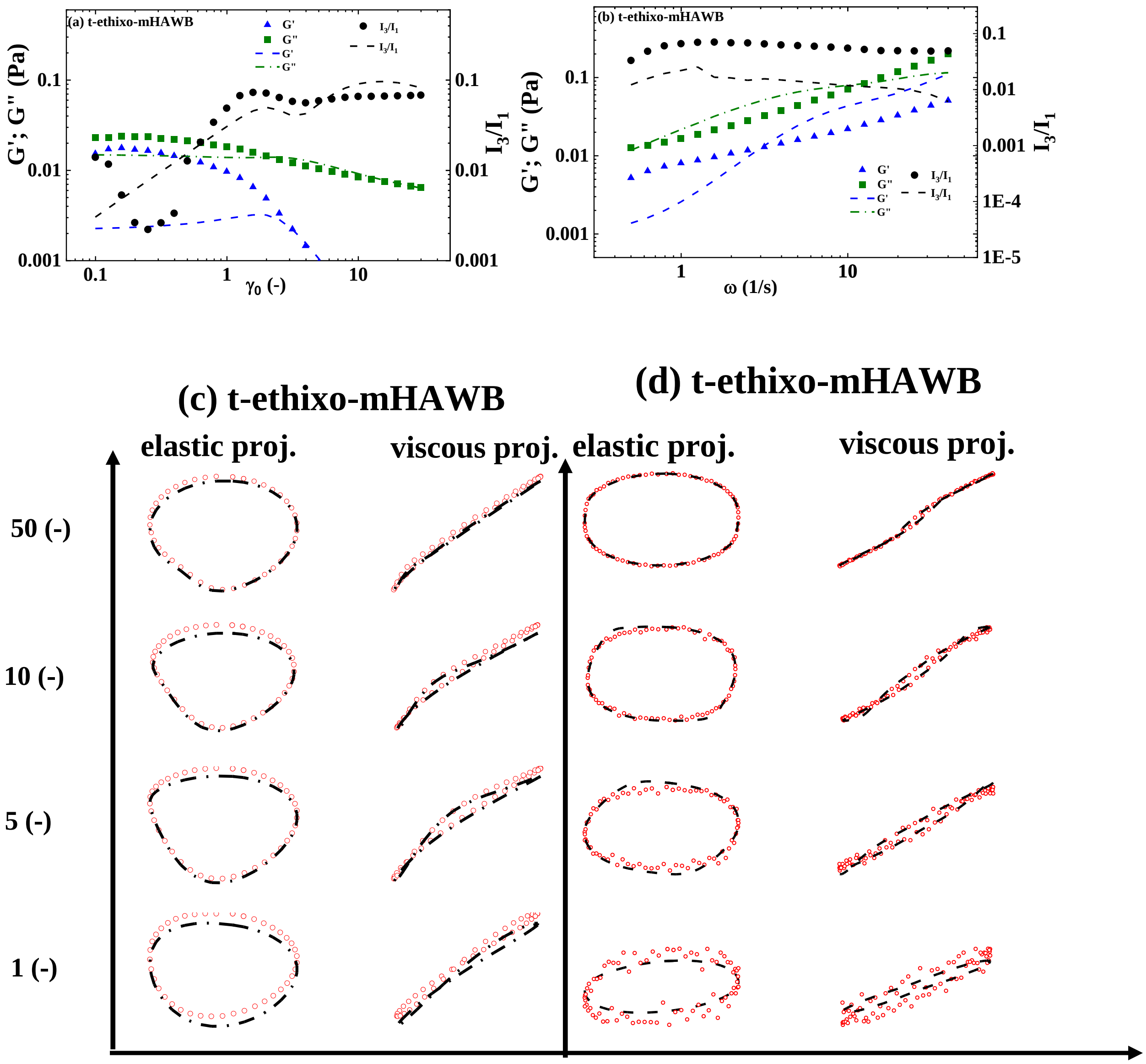

Figure S4. t-ethixo mHAWB prediction of (a) amplitude sweep executed at ω =12.566 (rad/s); (b) frequency sweep executed at γ_0=1(-); (c) elastic and viscous UDLAOS projections (green lines); and (d) elastic and viscous LAOS projections (green lines). (All UDLAOS and LAOS were conducted at ω =1 (rad/s), and corresponding strain amplitude). Red dots indicate data; x-axis units are frequency (rad/s); y-axis units are strain amplitude (-). (Donor 2, Dataset 2).[50]

Table SI. Best fit model parameters, and F_{cost} of fits for Donor 1 with t-e-TVP and t-ethixo-mHAWB models. Highlight legend: yellow – steady state fit; green - fixed; orange (salmon column) t-e-TVP step up/down fits; orange (blue column) - t-ethixo-mHAWB step up/down fits. (Donor 2, Dataset 2).[50]

	DONOR2	
**Par.**	**t-e-TVP**	**t-ethixo-mHAWB**
μ_0 (Pa s)	(-)	0.0051
μ_∞ (Pa s)	0.0038	0.0034
σ_{y0} (Pa)	0.0019	0.002060
t_{r1} (s)	0.2500	0.3928
t_{r2} ($s^{1/2}$)	0.0523	0.2056
μ_R or η_{ST} (Pa s)	0.0208	0.0201
τ_C (s)	(-)	0.0093
d	1/2	1/2
m	3/2	3/2
γ_∞	(-)	1
τ_λ (s)	0.4876	1.1278
G or G_R(Pa)	0.2735	0.1848
G_c (Pa)	(-)	0.2211
F_{cost} (SS)*	0.0292	0.0232
n (points)	16	16
RSS	0.2183	0.1374
k (par.)	5	7
AIC	6.9562	10.0308
BIC	24.6821	34.8471
F_{cost} (steps)*	0.000440	0.00042904
n (points)	390	390
RSS	0.0295	0.0280
k (par.)	7	10
AIC	6.9523	12.8487
BIC	76.4784	112.1717

Table S2. F_{cost} comparison for model predictions (SAOS, LAOS, UDLAOS) over experimental fits (steady state, shear rate step tests; note that steady state is not included because it is nondimensionalized) (Donor 2).

	**DONOR2**	
**F_{cost}**	**t-e-TVP**	**t-ethixo-mHAWB**
**SS***	**0.02920**	**0.02317**
**steps**	**0.00044**	**0.00043**
**Amp. Sweep**	**0.0011**	**0.0009**
**Freq. Sweep**	**0.0036**	**0.0040**
**UDLAOS**	**0.0043**	**0.0018**
**LAOS**	**0.0021**	**0.0006**
**Sum**	**0.01159**	**0.00770**

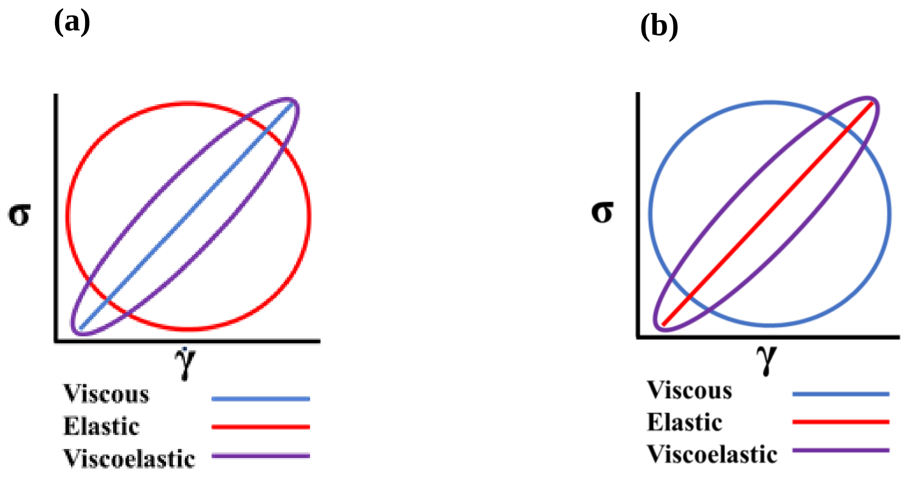

Figure S5: Legend for interpretation of: (a) viscous projection; and (b)elastic projection.

[50] Dataset4 M.J. Armstrong, J.S. Horner "JUL18 Human blood rheology", Mendeley, 2020. DOI: 10.17632/s8w6s6f68b.1.
